# Residual yolk depletion matches the establishment of functional oral-intestinal microbiota in hatchling Siamese crocodiles

**DOI:** 10.1016/j.isci.2026.117047

**Published:** 2026-08-04

**Authors:** Jun-Qiong Chen, Dan Liu, Qing-Yan Meng, Yun-Tao Yao, Chi-Xian Lin, Ying-Hao Shi, Yu Du, Yan-Fu Qu, Xiang Ji

**Affiliations:** 1 Hainan Key Laboratory for Herpetological Research, College of Fisheries and Life Science, Hainan Tropical Ocean University, Sanya 572022, China; 2 Zhejiang Provincial Key Laboratory for Water Environment and Marine Biological Resources Protection, College of Life and Environmental Sciences, Wenzhou University, Wenzhou 325035, China; 3 College of Life Sciences, Nanjing Normal University, Nanjing 210023, China; 4 Longze Agricultural Science and Technology Development Co., Ltd., Haikou 570125, China

**Keywords:** bacterial colonization, environmental microbiota, intestinal microbiota, oral microbiota, residual yolk, siamese crocodile

## Abstract

Newborn reptiles rely on residual yolk as their sole energy source before feeding, yet it is unknown whether functionally competent oral and intestinal microbial communities fully develop before yolk depletion. Using hatchling Siamese crocodiles (*Crocodylus siamensis*), we tested the hypothesis that yolk depletion aligns with the establishment of vital early-life oral and intestinal microbial communities. We profiled oral, intestinal, and environmental microbiomes over 56 post-hatching days with 16S rRNA gene sequencing. Egg-derived microbes acted as the initial colonizing inoculum. Bacterial diversification began soon after hatching. Bacterial colonization in the oral cavity and intestine was non-random and site-specific, with environmental and dietary correlates of microbiota composition being evident at both sites. Critically, pre-feeding oral and intestinal communities possessed phylogenetically diverse bacteria with broad functional capacity. Our results confirm that yolk depletion coincides with the formation of functionally competent oral and intestinal microbiota in hatchling crocodiles.

## Highlights

• Egg-derived microbes act as the initial colonizing inoculum for *C. siamensis*
• Oral and intestinal bacterial communities diversify soon after hatching
• Bacterial colonization in oral and intestinal tracts is nonrandom and site-specific
• Yolk depletion matches the establishment of functional oral-intestinal microbiota

## Graphical abstract

**Figure:**
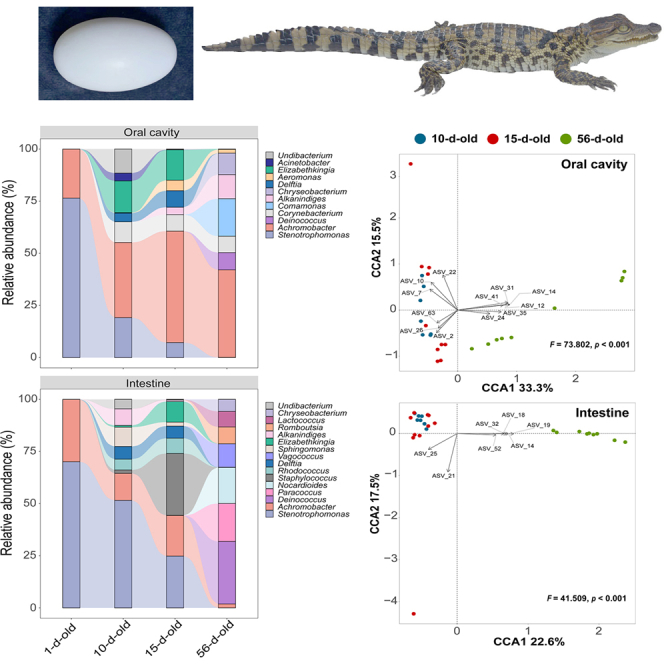

## Teaser

Microbiology; Oral-intestinal microbiota; Developmental biology

## Introduction

The assembly of oral and intestinal microbiota during early life is crucial, shaping developmental trajectories that bolster immunity, facilitate host-inaccessible digestive and metabolic functions, and exert long-term impacts on host health and fitness.^1^^,^^2^^,^^3^^,^^4^^,^^5^ For example, symbiotic gut bacteria aid in the degradation of complex polysaccharides (e.g., cellulose) and oligosaccharides^6^ and the biosynthesis of essential amino acids, thereby supporting host nutrition.^7^ Studies across multiple vertebrate taxa including fish,^8^ amphibians,^9^ reptiles,^2^^,^^10^^,^^11^ birds,^12^^,^^13^ and mammals^14^^,^^15^ demonstrate that microbiota assembly initiates before hatching (oviparous species) or birth (viviparous species) predominantly via maternal transmission and accelerates thereafter through rapid environmental and parental colonization. Consequently, the post-hatching or postnatal dynamics and stability of microbial communities, reflecting host-environment interactions, are a central focus in microbial ecology.^3^^,^^8^^,^^11^^,^^16^

In hatchling reptiles, the first days to weeks of life involve critical behavioral and physiological transitions that enable the establishment of a symbiotic gut microbiota, essential for augmenting foraging, immune, and digestive capacities (e.g., fibrous plant degradation in herbivores).^17^ For instance, newly hatched green iguanas (*Iguana iguana*) may spend approximately three weeks dispersing from nests and engaging in coprophagy to acquire bacteria essential for their intestinal fermentation system.^18^^,^^19^ Such as other reptiles,^17^^,^^20^ they rely on residual yolk (the portion of yolk remaining unused at hatching) as their sole energy source for maintenance metabolism and other essential activities, including somatic tissue growth until foraging begins and their intestinal fermentation system becomes fully functional.^19^^,^^21^ Notably, residual yolk depletion in green iguanas (3–4 weeks) slightly exceeds the time needed to establish a functional intestinal microbiota prior to first feeding.^19^^,^^21^ This temporal mismatch prompts a key question: Does residual yolk depletion coincide with the establishment of oral and intestinal microbial communities essential for early life? If so, we hypothesize that core microbial taxa colonize the oral and intestinal niches prior to residual yolk exhaustion. Testing this hypothesis requires longitudinal monitoring of microbiota dynamics across early life stages, spanning the time interval before and after residual yolk exhaustion, in a species with substantial residual yolk reserves.

Among reptiles, crocodilians possess the highest relative residual yolk reserves (*∼*33% of body dry mass), compared to lizards (∼6%), snakes (∼17%), and turtles (∼17%).^17^ We therefore selected the Siamese crocodile (*Crocodylus siamensis*) as our model system to test the above hypothesis. This species is native to Southeast Asia (Brunei, Cambodia, Indonesia including Borneo and possibly Java, Laos, Malaysia, Myanmar, Thailand, and Vietnam)^22^; it was introduced to China for aquaculture in the early 21st century.^23^ In our study population, residual yolk is exhausted within 15–18 days (mean ≈16 days).

To test our hypothesis, we analyzed oral and intestinal microbial samples (via 16S rRNA gene sequencing) collected from hatchlings on days 1, 10, 15, and 56 post-hatching—time points spanning the period before and after residual yolk exhaustion. Environmental samples (albumen and eggshell samples collected on the day of hatching; water and soil samples collected on days 10, 15, and 56 post-hatching) were concurrently analyzed to assess potential microbial sources.

## Results

### Environmental microbiota

**Figure 1:**
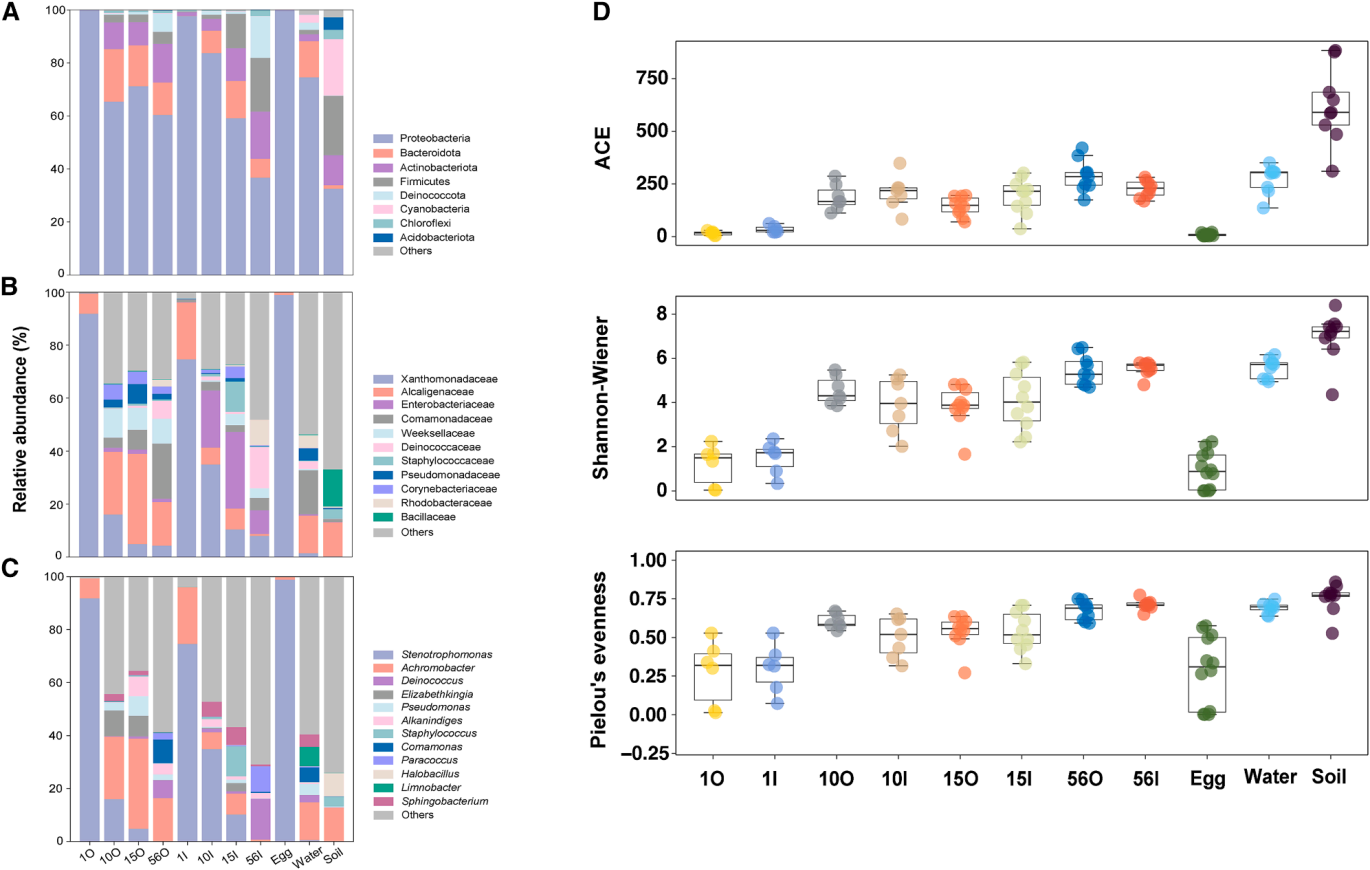
Oral, intestinal, and environmental microbiota The relative abundance of oral, intestinal, and environmental microbiota at the phylum (A), family (B), and genus (C) levels and box plots for three a-diversity indices (D) in each group of samples. 1O, 10O, 15O, and 56O indicate oral samples respectively collected from 1-, 10-, 15-, and 56-d-old hatchlings. 1I, 10I, 15I, and 56I indicate intestinal samples respectively collected from 1-, 10-, 15-, and 56-d-old hatchlings. Egg, Water, and Soil indicate environmental samples respectively collected from hatched eggs (albumen and inner eggshell), water, and soil. Each color in Plots A, B, and C indicates one taxonomic group, with its name given on the right side of each plot. The color for “others” in each plot indicates all other phyla (A), families (B), or genera (C) with a relative abundance <1%. Each color in Plot D represents one sample group; medians, upper and lower quartiles, and value ranges for all groups are displayed.

The composition of the microbiota did not differ significantly between eggshell and albumen samples based on Bray-Curtis [analysis of similarity (ANOSIM): *R* = −0.15, *p* = 0.97; permutational multivariate analysis of variance (PERMANOVA): *F*_1,10_ = 0.01, *p* = 0.92] or weighted UniFrac (ANOSIM: *R* = −0.09, *p* = 1.00; PERMANOVA: *F*_1,10_ = 0.02, *p* = 0.87) distances, nor did the relative abundance of the microbiota (paired-sample *t* test; all *t* < 0.74 and *p* > 0.47). Therefore, we pooled data by egg and classified eggshell and albumen samples as the environmental samples for 1-day-old hatchings, in which the dominant taxa were the phylum Proteobacteria (99.9%, Figure 1A), the family Xanthomonadaceae (98.9%, Figure 1B), and the genus *Stenotrophomonas* (99.1%, Figure 1C).

None of the three microbial α-diversity indices differed among water or soil samples collected on days 10, 15, and 56 post-hatching (all *H*_2,9_ < 5.96 and *p* > 0.50; Figure 1D and Table S1). Therefore, we pooled data by the medium (water or soil) and considered water and soil samples collected at the above three time points as the environmental samples for 10-, 15-, and 56-day-old hatchlings. Abundance-based coverage estimator (ACE) (*t* = 5.40, *df* = 16, *p* < 0.001) and Shannon-Wiener (*t* = 3.52, *df* = 16, *p* < 0.01) indices differed between water and soil samples, but the Pielou evenness index did not (*t* = 1.80, *df* = 16, *p* = 0.091) (Figure 1D). The dominant phyla (>10% relative abundance) were Proteobacteria (74.6%) and Bacteroidota (13.7%) in water samples, versus Proteobacteria (32.5%), Firmicutes (22.4%), Cyanobacteria (21.4%), and Actinobacteriota (11.3%) in soil samples (Figure 1A). The dominant families (>10% relative abundance) included Comamonadaceae (15.6%) and Alcaligenaceae (14.3%) in water samples, and Bacillaceae (14.0%) and Alcaligenaceae (12.9%) in soil samples (Figure 1B). *Achromobacter* (phylum Proteobacteria) was the sole dominant bacterial genus (10% relative abundance) in both water (14.3%) and soil (12.9%) samples (Figure 1C).

### Oral and intestinal microbiota

Microbial composition was similar between the oral cavity and intestine at hatching, with the phylum Proteobacteria (Figure 1A), the families Xanthomonadaceae and Alcaligenaceae (Figure 1B), and the genera *Stenotrophomonas* and *Achromobacter* (Figure 1C) dominating at both sites in 1-day-old hatchlings. Diversification of oral and intestinal microbial communities commenced shortly after hatching: By day 56, dominant oral phyla (>10% mean relative abundance) were Proteobacteria (60.4–71.3%), Bacteroidota (12.3–19.8%), and Actinobacteriota (8.8–14.6%), while dominant intestinal phyla included Proteobacteria (36.7–83.8%), Firmicutes (1.6–20.3%), Deinococcota (0.83–15.8%), Actinobacteriota (4.4–17.8%), and Bacteroidota (7.2–14.6%) (Figure 1A). In hatchlings aged ≥10 days, only one genus (*Achromobacter* 16.4–34.0%) and 4 families (Alcaligenaceae 16.4–34.1%, Xanthomonadaceae 4.3–16.1%, Weeksellaceae 8.3–10.8%, and Comamonadaceae 3.8–20.8%) were dominant in the oral cavity, and 3 genera (*Deinococcus* 0.83–15.5%, *Staphylococcus* nil-11.3%, and *Stenotrophomonas* 0.01–35.0%) and 4 families (Deinococcaceae 0.83–15.5%, Enterobacteriaceae 8.9–28.9%, Xanthomonadaceae 8.1–35.0%, and Staphylococcaceae 0.32–11.4%) were dominant in the intestine (Figures 1B and 1C).

Mantel tests revealed no significant oral-intestinal microbiota correlations in hatchlings at any age (|*r*| < 0.29 and *p* > 0.13 in all cases). Paired-sample *t* tests showed: (1) none of the three α-diversity indices differed between the oral and intestinal microbiota in 56-day-old hatchlings (all *p* > 0.11; Figure 1D and Table S1); (2) ACE was higher in the intestine than in the oral cavity in 1- (*t* = 3.62, *df* = 5, *p* < 0.02) and 15-day-old hatchlings (*t* = 2.33, *df* = 9, *p* < 0.05) (Figure 1D and Table S1); (3) Shannon-Wiener index was higher in the intestine than in the oral cavity in 1-day-old hatchlings (*t* = 2.79, *df* = 5, *p* = 0.04; Figure 1D and Table S1); (4) Pielou evenness index was higher in the oral cavity than in the intestine in 10-day-old hatchlings (*t* = 2.75, *df* = 6, *p* = 0.03; Figure 1D and Table S1). The three α-diversity indices differed significantly with age for both the oral cavity and the intestine (Kruskal-Wallis test: all *H* > 14.00 and *p* < 0.01; Figure 1D and Table S1).

**Figure 2:**
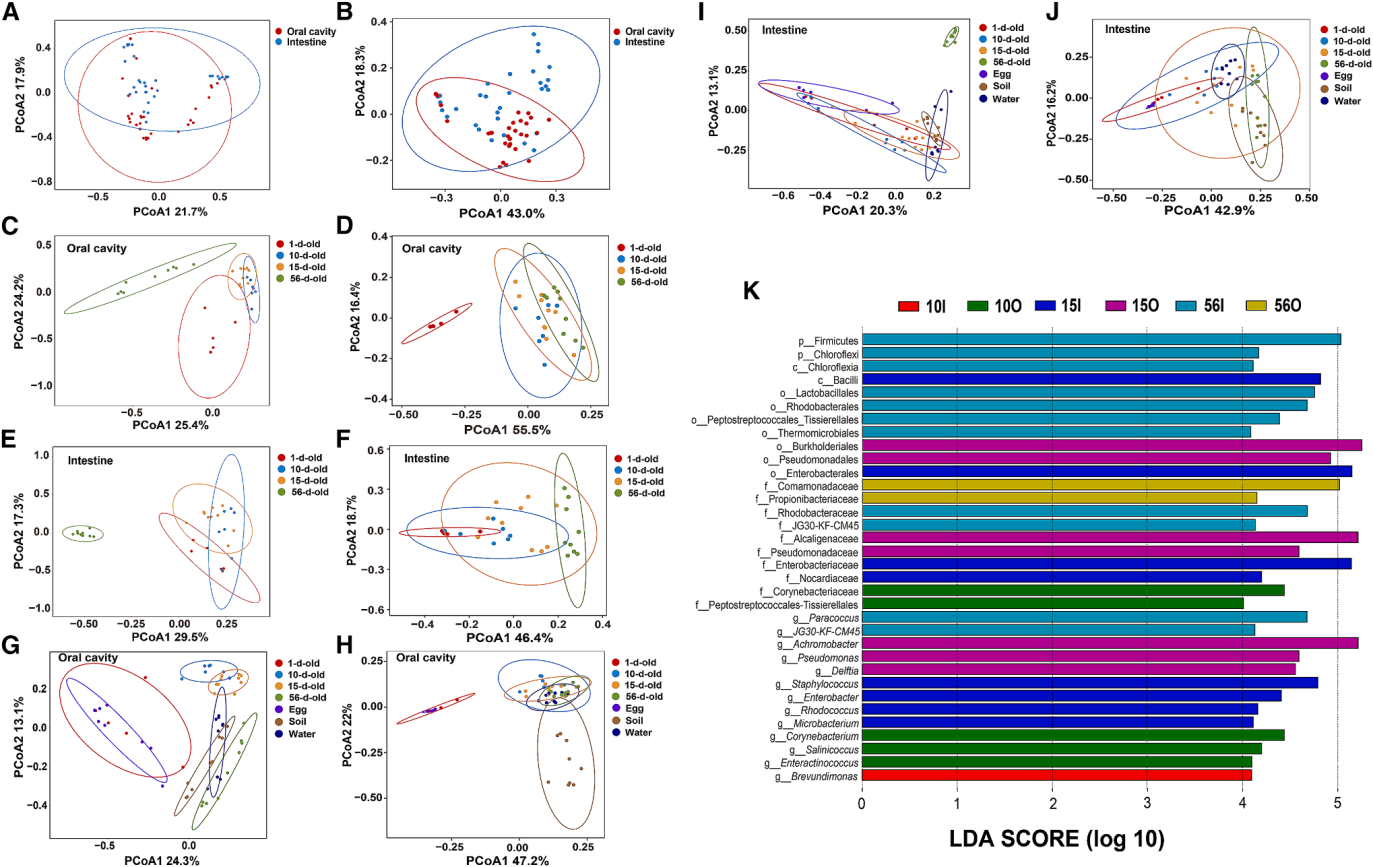
Principal coordinates analysis (PCoA) of microbial community composition PCoA plots are based on Bray–Curtis (A, C, E, G, and I) and weighted UniFrac (B, D, F, H, and J) distances. Overall oral and intestinal microbial diversity across all hatchlings (A and B), and oral and intestinal microbial diversity at different ages (days 1, 10, 15, and 56; C, D, E, and F); comparison of oral and intestinal microbial communities with those of potential environmental sources (water, soil, and egg; G, H, I, and J). Linear discriminant analysis (LDA) scores indicate oral and intestinal microbial markers at different days of age (LDA >4, *p* < 0.05) (K). Letters p, c, o, f, and g indicate phylum, class, order, family, and genus, respectively. See Figure 1 for definitions for 10O, 15O, 56O, 10I, 15I, and 56I.

Principal coordinates analysis (PCoA) based on Bray-Curtis and weighted UniFrac distances revealed a significant oral-intestinal separation (ANOSIM: both *R* > 0.14 and *p* < 0.001; PERMANOVA: both *F*_1,16_ > 5.10 and *p* < 0.001), with the first two coordinates cumulatively explaining 39.6% (21.7% on PCoA1, and 17.9% on PCoA2; Figure 2A) and 61.3% (43.0% on PCoA1, and 18.3% on PCoA2; Figure 2B) of total variance, respectively. Age-related separations were significant for oral (ANOSIM: both *R* > 0.66 and *p* < 0.001; PERMANOVA: both *F*_3,28_ > 8.74 and *p* < 0.001; Figures 2C and 2D) and intestinal (ANOSIM: both *R* > 0.59 and *p* < 0.001; PERMANOVA: both *F*_3,28_ > 6.89, *p* < 0.001; Figures 2E and 2F) microbiota, with the first two coordinates cumulatively explaining ≥47% of total variance (Figures 2C–2F).

LEfSe identified 34 unique bacterial markers at the phylum, class, order, family, and genus levels (Figure 2K). Mann-Whitney tests confirmed 14 unique bacterial taxa with a higher relative abundance (Table S2), including the genus *Brevundimonas* (10-day-old hatchlings), the class Bacilli, the order Enterobacterales, the families Nocardiaceae and Enterobacteriaceae, and the genera *Microbacterium*, *Rhodococcus*, and *Staphylococcus* (15-day-old hatchlings), the orders Peptostreptococcales_Tissierellales, Lactobacillales, and Rhodobacterales, the family Rhodobacteraceae, and the genus *Paracoccus* (56-day-old hatchlings) in the intestine, and the family Propionibacteriaceae (56-day-old hatchlings) in the oral cavity (Figure 2K and Table S2).

### Microbial source tracking and assembly dynamics

**Figure 3:**
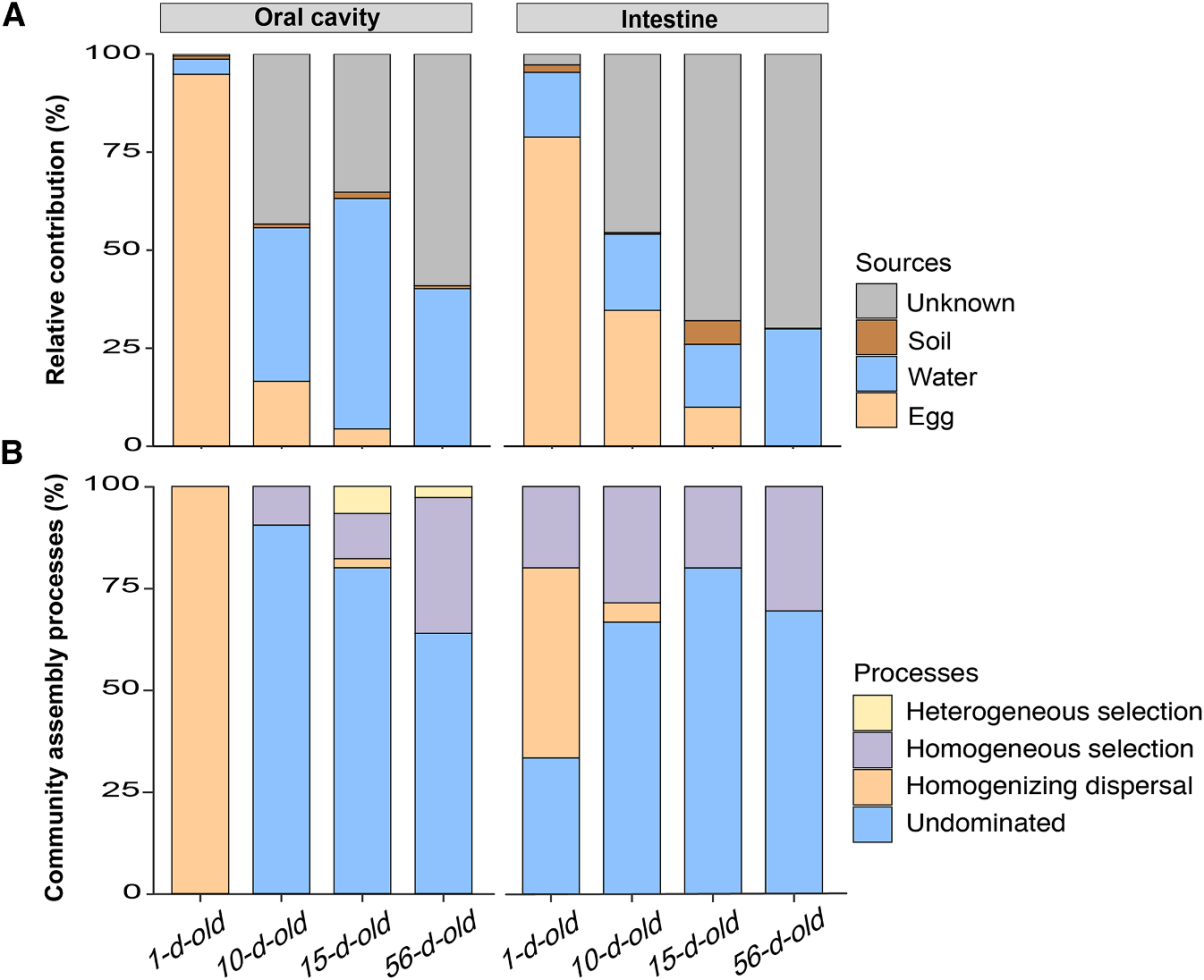
Microbial source tracking and assembly dynamics The relative contribution of the environmental (soil, water, and egg) microbiota to the oral and intestinal microbiota (A) and shifts in community assembly process (B) during the first 56 days post-hatching. Deterministic processes = heterogeneous selection + homogeneous selection; stochastic processes = homogenizing dispersal + undominated processes.

Bayesian source tracking (SourceTracker2) quantitatively revealed a clear successional shift in the contributions of potential microbial sources to the hatchling microbiota over the first 56 days post-hatching (Figure 3A). At hatching (day 1), the hatchling-associated microbial communities exhibited a predominantly egg-associated signature. The oral microbiota was predominantly derived from egg sources (94.8%), whereas the intestinal microbiota comprised a mixture of egg sources (78.8%) and external environmental sources (water and soil, collectively contributing 18.4%). However, upon transitioning to the pre-feeding stage (days 10 and 15), the contribution of the initial egg-associated sources declined rapidly. By day 15, the egg-associated contribution to the oral microbiota had decreased to 4.4%, surpassed by environmental sources (water and soil combined, 60.3%), with water being the dominant contributor. Concurrently, the egg-derived proportion in the intestinal microbiota decreased to 10.0%, while the proportion of unknown sources increased substantially to 68.0%, suggesting pronounced internal microbial diversification prior to the onset of exogenous feeding. At day 56, unknown sources represented the majority of both the oral (59.1%) and intestinal (69.9%) microbiota, with the remainder (30.1%–40.9%) attributed to environmental (water and soil) sources (Figure 3A). Null model-based analysis revealed temporal shifts in the dominant ecological processes governing hatchling microbiota assembly (Figure 3B). At hatching, oral microbiota assembly was governed entirely by homogenizing dispersal (100%). In contrast, intestinal assembly was driven by multiple processes: homogenizing dispersal (46.7%), undominated processes (33.3%), and homogeneous selection (20.0%). During the pre-feeding stage (days 10–15), undominated processes (ecological drift) became increasingly dominant in the assembly in both niches, accounting for 80.0–90.5% in the oral cavity and 66.7–80.0% in the intestine. Concurrently, homogeneous selection exhibited an increasing influence with host age, a trend that was particularly evident in the intestine, reaching 30.6% by day 56. Deterministic processes (homogeneous and heterogeneous selection combined) accounted for 36.1% of oral and 30.6% of intestinal assembly by day 56, although undominated processes remained the predominant driver (>60%) (Figure 3B).

### Temporal changes in core microbes

**Figure 4:**
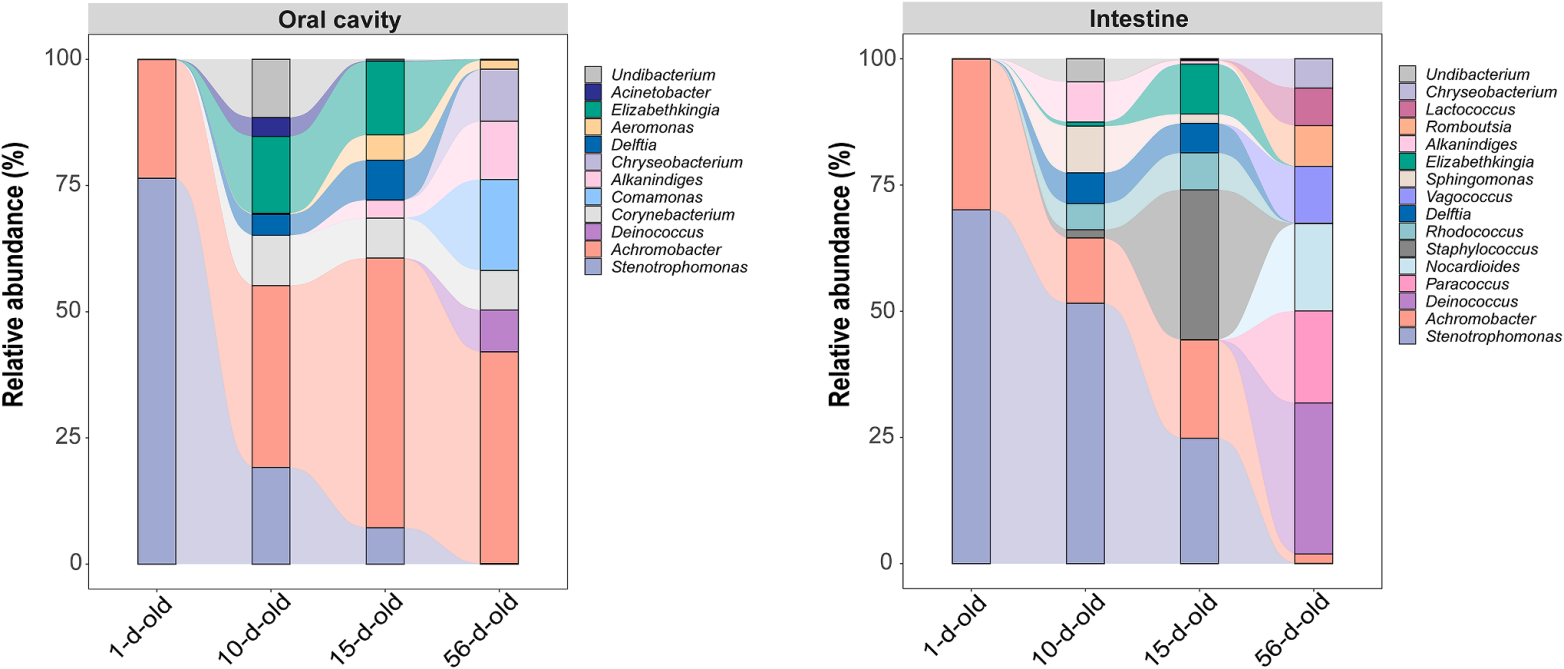
Temporal changes in core microbes Alluvial diagrams showing dynamic changes in the relative abundance of dominant oral (left) and intestinal (right) bacterial genera (detected in 90% of all oral or intestinal samples and with a mean relative abundance >2%) in the first 56 days after hatching. Each color indicates one genus. Relative abundance was re-normalized to 100% based on the union of core ASVs. See Figure 1 for definitions for 1O, 10O, 15O, 56O, 1I, 10I, 15I, and 56I.

On day 1, the core oral and intestinal microbes were identical but diverged thereafter (Figure 4). Bacteria of the genus *Stenotrophomonas* progressively decreased and were absent from the oral cavity and intestine by day 56 post-hatching, whereas bacteria of the genus *Achromobacter* persisted throughout this period (Figure 4). Post-hatching microbial colonization was site-specific: by day 56 post-hatching, core oral genera included *Corynebacterium* of the phylum Actinobacteriota, *Elizabethkingia* and *Chryseobacterium* of the phylum Bacteroidota, *Acinetobacter*, *Aeromonas*, *Delftia*, *Alkanindiges*, *Comamonas* and *Achromobacter* of the phylum Proteobacteria, and *Deinococcus* of the phylum Deinococcota (Figure 4). Core intestinal genera additionally included *Lactococcus*, *Romboutsia*, *Vagococcus*, and *Staphylococcus* of the phylum Firmicutes, *Sphingomonas* and *Paracoccus* of the phylum Proteobacteria, and *Rhodococcus* and *Nocardioides* of the phylum Actinobacteriota (Figure 4).

### Environmental correlates

**Figure 5:**
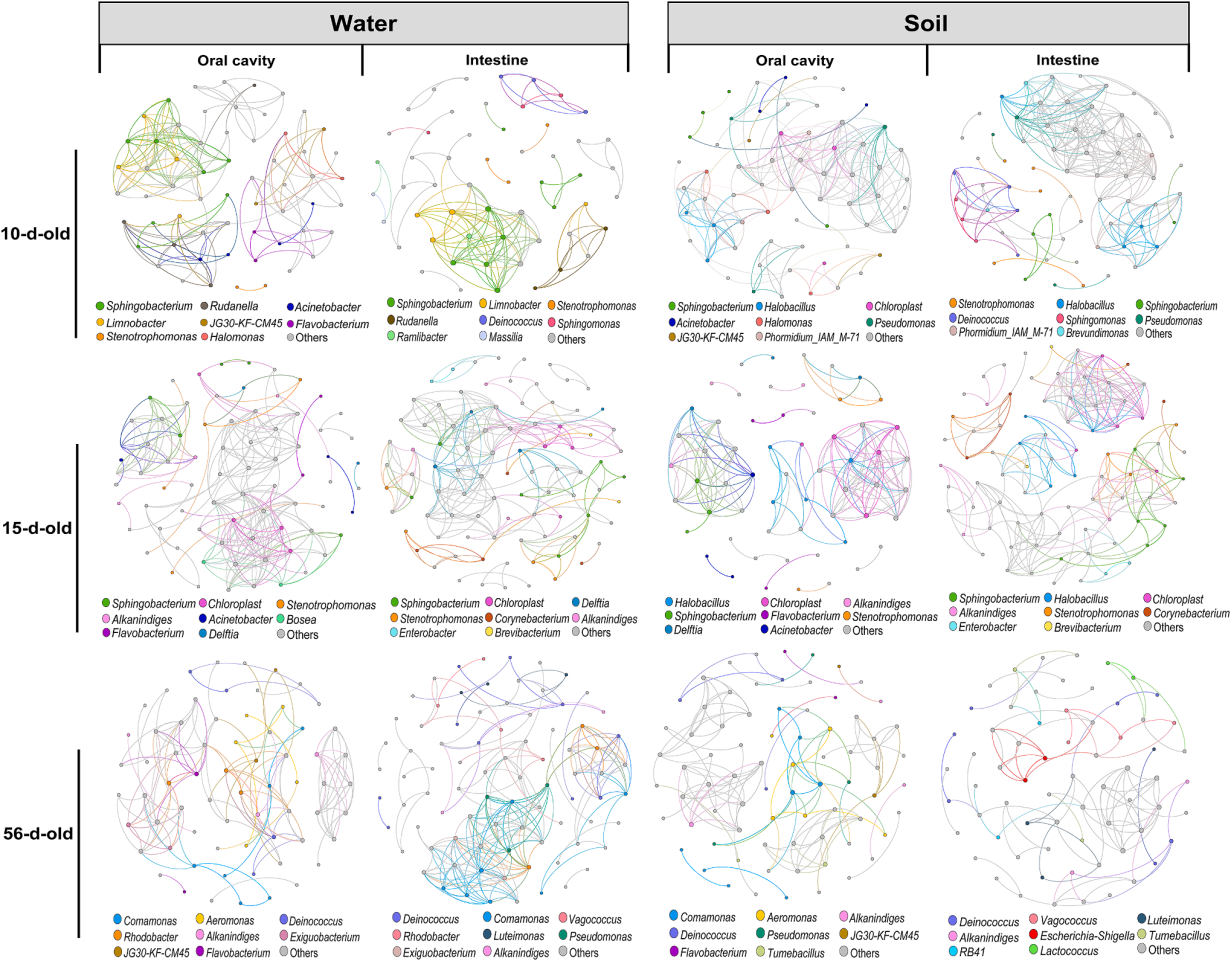
Co-occurrence networks of the oral and intestinal microbiota of 10-, 15-, and 56-d-old hatchlings and their environmental (water or soil) microbiota Only ASVs with a mean relative abundance >1% were included in analysis. Each color indicates one bacterial genus, and node size indicates the degree of correlation between ASVs. See Table S3 for network topological parameters.

ANOSIM revealed no significant differences between the oral/intestinal microbiota and the environmental (egg) microbiota at hatching (all *R* < 0.06, and all *p* > 0.205; Figures 2G–2J). PCoA further illustrated the successional trajectories of hatchling microbiota relative to potential sources. Both Bray-Curtis and weighted UniFrac distances showed that oral and intestinal samples from different ages formed distinct clusters separating from environmental and egg samples (Figures 2G–2J). Significant dissimilarities in community composition emerged in hatchlings aged ≥10 days (*R* > 0.67 and *p* < 0.012 in all cases), while significant differences in α-diversity indices were observed in 10- and 15-day-old hatchlings (Table S1). At hatching, genera such as *Stenotrophomonas*, *Delftia*, and *Achromobacter* (phylum Proteobacteria) dominated the shared nodes between the environmental (egg) and oral microbiota (Figure S1A), whereas *Cutibacterium* (phylum Actinobacteriota) also contributed to these shared nodes (Figure S1B). Network complexity increased with age. By day 10 post-hatching, genus-level nodes shared between soil and the oral/intestinal niches were predominantly composed of bacteria of the phyla Bacteroidota, Firmicutes, and Proteobacteria, whereas nodes shared between water and the oral/intestinal niches involved bacteria of the phyla Bacteroidota and Proteobacteria (Figure 5 and Table S3). The composition of soil-intestinal shared nodes at the genus level differed slightly between days 10 and 15, with the day 15 nodes additionally comprising amplicon sequence variants (ASVs) assigned to the phylum Actinobacteriota (Figure 5). Similarly, genus-level water-oral shared nodes included ASVs assigned to the phylum Cyanobacteria, while water-intestinal shared nodes included ASVs from the phyla Cyanobacteria and Actinobacteriota (Figure 5). By day 56 post-hatching, genus-level nodes shared between environmental (water and soil) sources and the oral or intestinal microbiota were primarily composed of bacteria of the phyla Proteobacteria, Deinococcota, and Firmicutes (Figure 5). Although the phylum-level composition of nodes shared between environmental sources and host-associated niches was similar, the genus-level structure of the oral and intestinal microbiota themselves diverged significantly in hatchlings aged ≥10 days (Figure 5).

**Figure 6:**
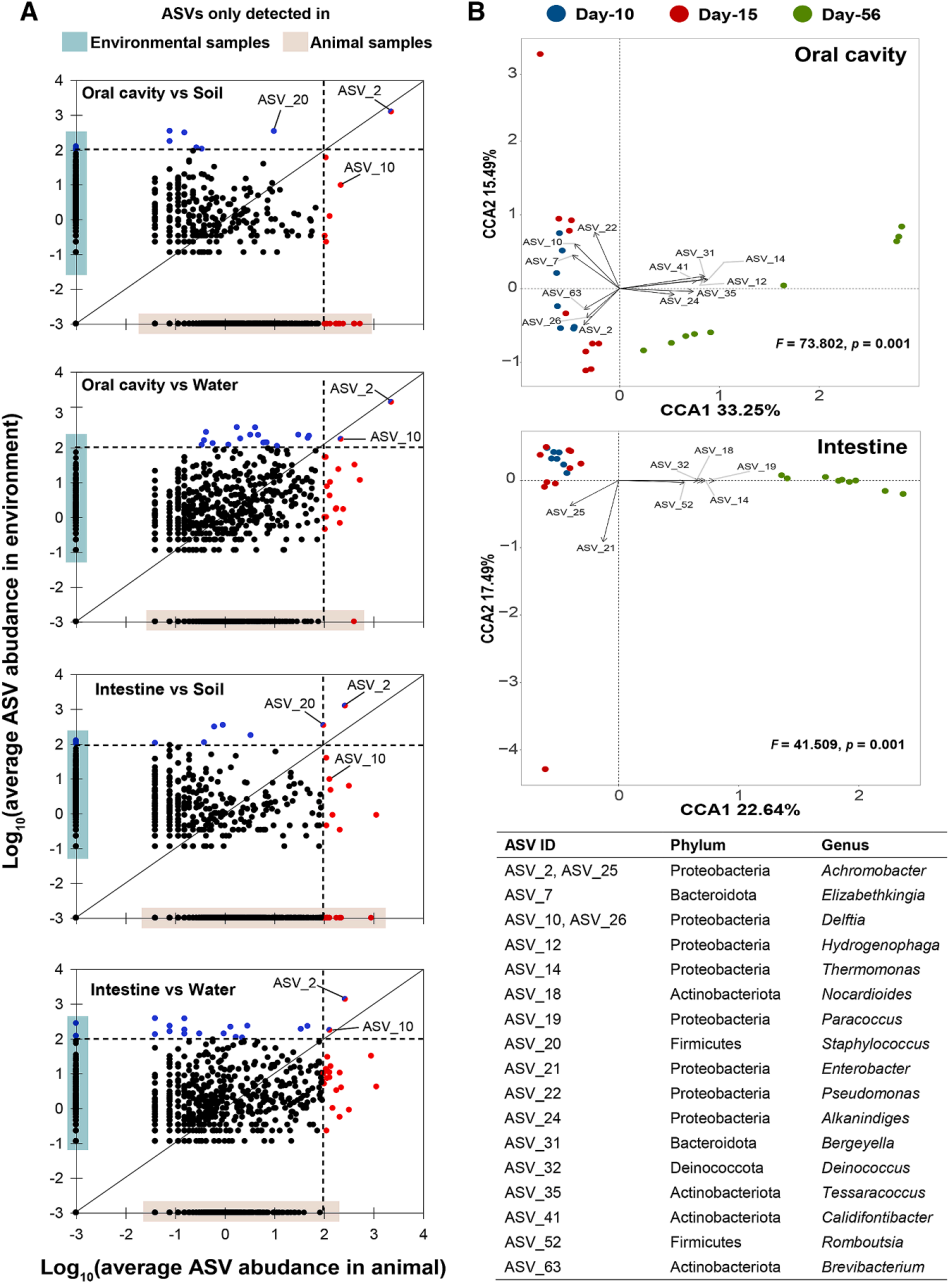
Environmental correlates of oral and intestinal microbiota The mean relative abundance of each bacterial ASV detected in host animals (oral cavity or intestinal tract) and their external environment (water or soil) (A) and variation in oral and intestinal microbial communities explained by the top 20 ASVs (B). Plot A also shows the position of each host-environment pair of ASVs relative to the diagonal line. The dashed line represents a mean relative abundance of 1%. Animal (oral cavity and intestine) and environmental (water and soil) ASVs with a mean relative abundance >1% are in red and blue, respectively; animal and environmental ASVs with a relative abundance ≤1% are in black. Red/blue bi-colored dots indicate dominant ASVs in the oral cavity, intestine, and/or environment. Dots in Plot B indicate individual oral or intestinal bacterial communities at different days of age, and arrows in the plot indicate the relative contributions of the top 20 bacterial ASVs.

Oral and intestinal microbiota diverged from environmental microbiota in the first 56 days of life (Figure 6). For the ASVs with a relative abundance >1%, only oral ASV2 (*Achromobacter* of the phylum Proteobacteria) and intestinal ASV2 and ASV20 (*Staphylococcus* of the phylum Firmicutes) overlapped with soil samples, and ASV2 and ASV10 (*Delftia* of the phylum Proteobacteria) detected in oral and intestinal samples overlapped with water samples. Most other relatively prevalent ASVs in oral or intestinal samples were not abundant in environmental samples (Figure 6A). Canonical correspondence analysis (CCA) indicated that the top 20 oral and intestinal ASVs cumulatively explained 48.7% (oral) and 40.1% (intestinal) of community variation in the first two vectors (Figure 6B). Oral and intestinal microbial samples collected before (days 10 and 15 post-hatching) and after (day 56 post-hatching) feeding were completely separated along the first two CCA vectors, whereas oral and intestinal samples collected from 10- to 15-day-old hatchlings were partly separated (Figure 6B). The contributions of individual dominant ASVs differed between the oral and intestinal microbiota (Figure 6B). By day 10 post-hatching, the dominant bacterial taxa in the oral cavity were ASV2, ASV7 (*Elizabethkingia* of the phylum Bacteroidetes), ASV10, and ASV26 (*Delftia*), while none of the bacterial taxa was dominant in the intestine. By day 15 post-hatching, the oral cavity was dominated by ASV22 (*Pseudomonas* of the phylum Proteobacteria) and ASV63 (*Brevibacterium* of the phylum Actinobacteriota), and the intestine was dominated by ASV21 (*Enterobacter* of the phylum Proteobacteria) and ASV25 (*Achromobacter* of the phylum Proteobacteria). By day 56 post-hatching, the oral cavity and intestine were colonized by more dominant bacterial taxa: ASV12 (*Hydrogenophaga* of the phylum Proteobacteria), ASV14 (*Thermomonas* of the phylum Proteobacteria), ASV24 (*Alkanindiges* of the phylum Proteobacteria), ASV31 (*Bergeyella* of the phylum Bacteroidota), ASV35 (*Tessaracoccus* of the phylum Actinobacteriota), and ASV41 (*Calidifontibacter* of the phylum Actinobacteriota) in the oral cavity; ASV14, ASV18 (*Nocardioides* of the phylum Actinobacteriota), ASV19 (*Paracoccus* of the phylum Proteobacteria), ASV32 (*Deinococcus* of the phylum Deinococcota), and ASV52 (*Romboutsia* of the phylum Firmicutes) in the intestine.

### Temporal dynamics

**Figure 7:**
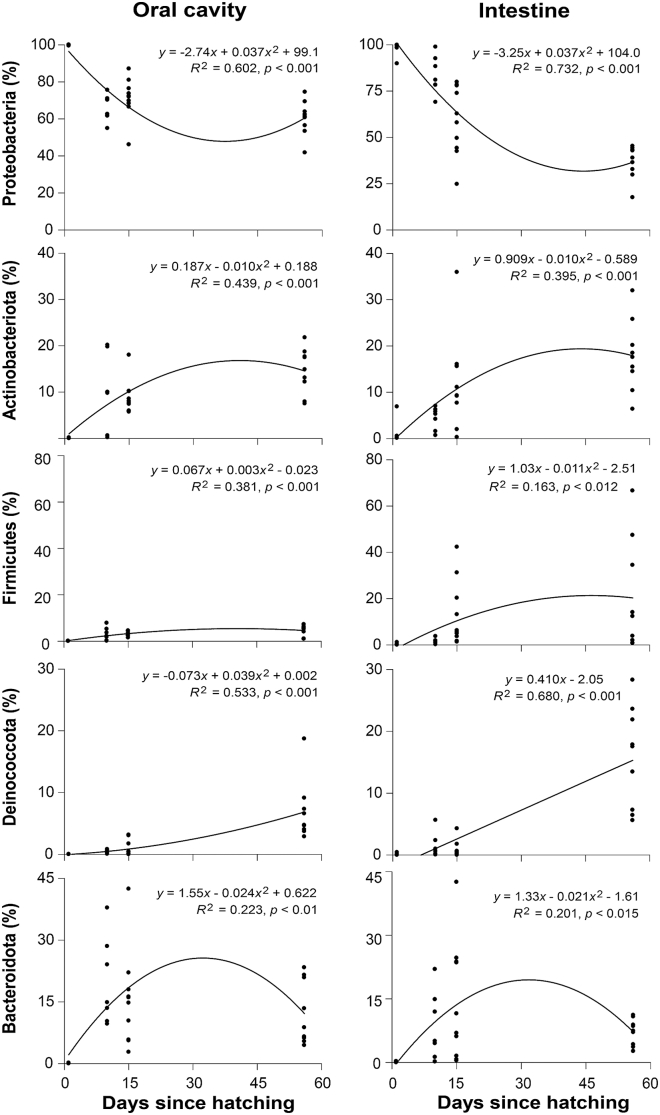
Regression curves or lines describing dynamic changes in relative abundance of the top five dominant oral (left five plots) and intestinal (right five plots) bacterial phyla in the first 56 days after hatching Regression equations, coefficients, and significance levels are given in the figure.

**Figure 8:**
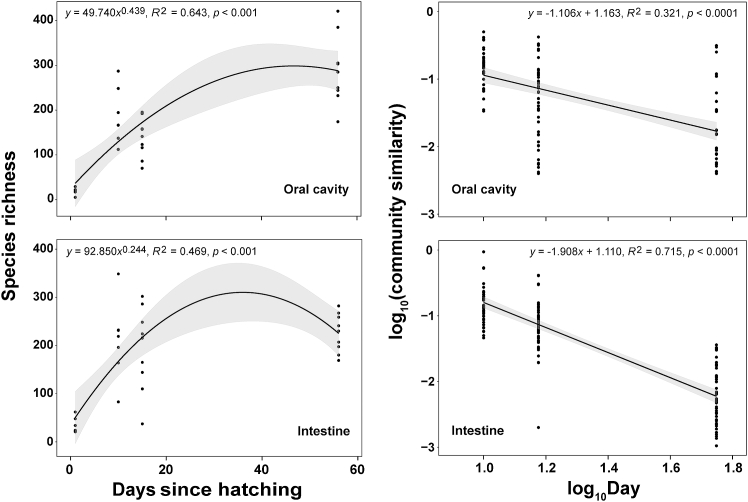
Regression curves or lines describing dynamic changes in species richness and community similarity of oral (upper two plots) and intestinal (lower two plots) microbiota in the first 56 days after hatching Regression equations, coefficients, and significance levels are given in the figure.

Five oral and intestinal bacterial phyla (>1% relative abundance) exhibited similar temporal trends: four (Actinobacteriota, Firmicutes, Deinococccota, and Bacteroidota) generally increased, while one (Proteobacteria) decreased during the first 56-day period (Figure 7). Species-time relationship (STR) analysis confirmed increasing species richness (both *p* < 0.001), with faster accumulation in the oral cavity (exponent = 0.439) than in the intestine (exponent = 0.244) (Figure 8A). Time-decay relationship (TDR) models estimated by logarithmic microbial similarity based on 1 minus Bray-Curtis distance showed that the rate (regression slope) at which the microbial community changed over time was −1.106 in the oral cavity and −1.908 in the intestine (Figure 8B). STR and TDR both suggested rapid microbial succession in the oral cavity and intestine in the early post-hatching days.

**Figure 9:**
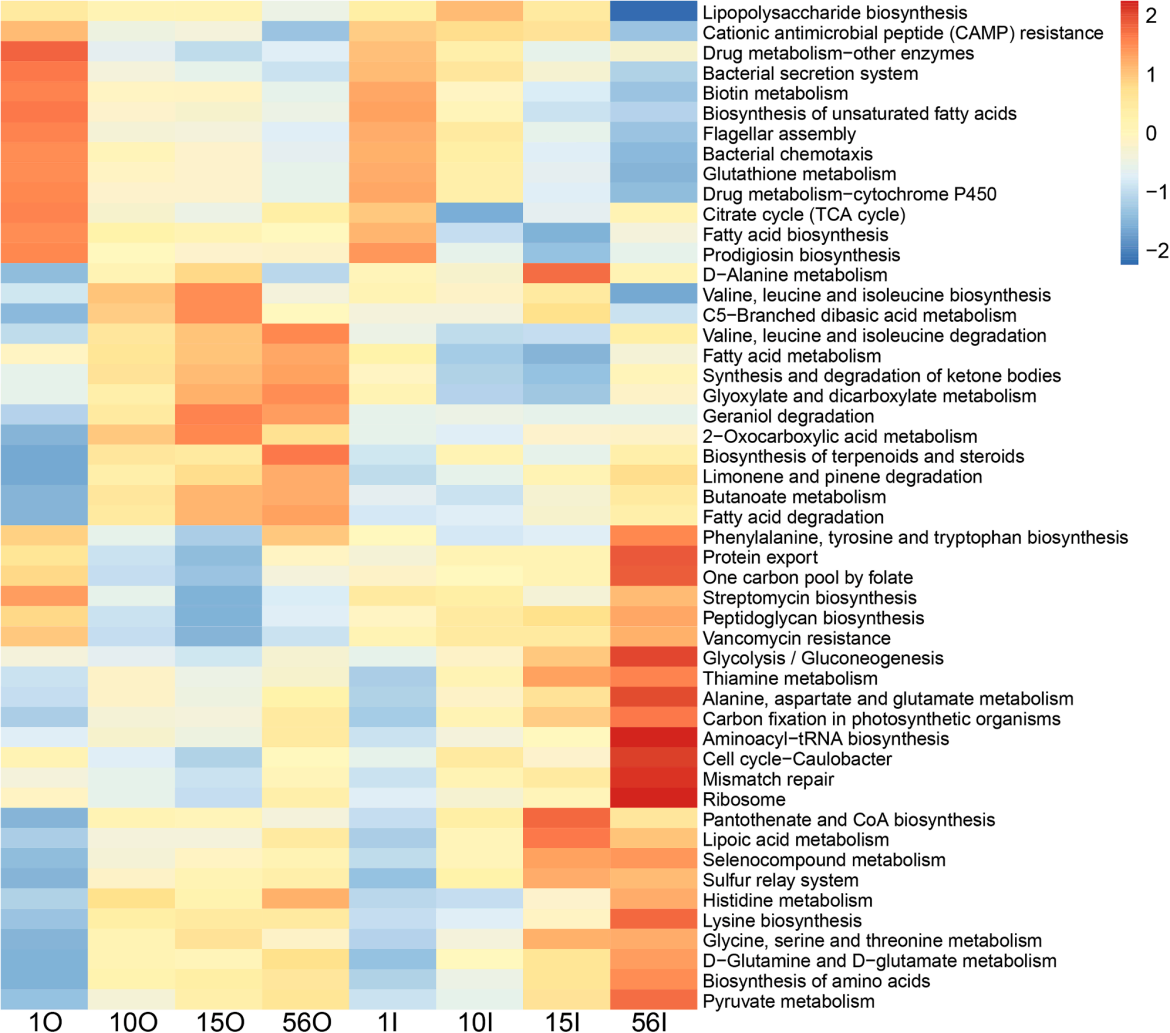
A heatmap showing the top 50 predicted KEGG (level 3) functions of oral and intestinal microbial communities at different days of age Colors in individual cells represent the *Z* score values of phenotype abundance. See Figure 1 for definitions for 1O, 10O, 15O, 56O, 1I, 10I, 15I, and 56I.

Functional predictions revealed that the oral and intestinal microbiota in 1-day-old hatchlings included taxa linked to functions such as lipopolysaccharide biosynthesis, cationic antimicrobial peptide resistance, drug metabolism-other enzymes, bacterial secretion system, biotin metabolism, unsaturated fatty acid biosynthesis, flagellar assembly, bacterial chemotaxis, glutathione metabolism, and citrate cycle (Figure 9). In hatchlings aged ≥10 days, oral microbial functions shifted to valine, leucine and isoleucine degradation, fatty acid metabolism, synthesis and degradation of ketone bodies, glyoxylate and dicarboxylate metabolism, biosynthesis of terpenoids and steroids, limonene and pinene degradation, butanoate metabolism, and fatty acid degradation, while intestinal microbial functions shifted to peptidoglycan biosynthesis, vancomycin resistance, glycolysis, gluconeogenesis, thiamine metabolism, alanine, aspartate, and glutamate metabolism, cell cycle-Caulobacter, pantothenate and CoA biosynthesis, lipoic acid metabolism, and selenocompound metabolism (Figure 9).

## Discussion

The principal findings of this study can be summarized as follows: (1) Bacterial communities in the external environment (soil and water) exhibited substantially greater complexity than those within the egg and remained largely unchanged throughout the study period, spanning from hatching to day 56 post-hatching (Figure 1). (2) Despite significant environmental correlates of the oral and intestinal microbiota (Figure 5), post-hatching bacterial colonization in these sites was nonrandom and selective, as evidenced by the low abundance of the most prevalent oral and intestinal ASVs in the external environment (Figure 6A). (3) Colonization of new core bacteria in the oral cavity and intestine was site-specific in hatchling *C. siamensis*, as indicated by the absence of significant intercorrelation between oral and intestinal microbiota at any time point during the first 56 days post-hatching. (4) Core bacteria in the oral cavity and intestine were nearly identical at hatching and became quite different thereafter; genus-level oral and intestinal microbiota underwent substantial restructuring by day 10 post-hatching, with the intestinal tract harboring significantly more core bacteria by day 56 post-hatching. (5) Hatchlings before and after first feeding were completely separated in the CCA ordination space defined by the top 20 dominant oral or intestinal ASVs, whereas 10- and 15-day-old hatchlings exhibited partial separation (Figure 6B). These findings collectively support the following conclusions.

First, egg-associated microbes constitute the primary inoculum for the early-life oral and intestinal microbiota of *C. siamensis*, encompassing taxa associated with foundational functions such as drug metabolism and cationic antimicrobial peptide resistance (Figure 9). Although our results demonstrate a high degree of similarity between the egg and hatchling microbiota, this pattern does not differentiate between maternal and environmental sources. This initial inoculum likely represents a composite derived from both maternal transmission and environmental colonization. In oviparous reptiles that lack parental care, the internal egg microbiota may originate from nest substrates, the maternal cloaca, and/or maternal oviducts.^2^^,^^3^^,^^10^^,^^24^ However, in species where females guard their nests, such as *C. siamensis*, the maternal skin and fecal microbiota may serve as additional sources of microbial taxa.^25^ The predominance of *Stenotrophomonas* bacteria (phylum Proteobacteria; family Xanthomonadaceae) in the oral cavity and intestine of 1-day-old hatchlings contrasts with the findings in hatchling striped plateau lizards (*Sceloporus virgatus*), in which the gastrointestinal microbiota is dominated by bacteria of the families Enterobacteriaceae and Yersiniaceae (both within the phylum Proteobacteria), reportedly primarily derived from maternal oviducts.^2^ This discrepancy suggests that the relative importance of distinct microbial colonization routes—such as pre-ovipositional maternal inoculation versus post-ovipositional environmental acquisition via the eggshell—varies among reptilian species. Bacteria of the genus *Stenotrophomonas* exhibit context-dependent roles,^26^^,^^27^^,^^28^^,^^29^ functioning as opportunistic pathogens in immunocompromised hosts^27^^,^^28^ or as beneficial symbionts conferring heavy metal and antibiotic resistance, participating in nitrogen and sulfur cycling, and facilitating bioremediation via the degradation of diverse compounds, including pollutants.^29^ Their absence in 56-day-old hatchlings (Figure 4) suggests a transient, early-life role, which may be particularly critical for crocodilian species such as *C. siamensis* that inhabit aquatic-terrestrial ecotones.

Second, major microbial taxa have colonized the oral and intestinal niches prior to residual yolk depletion. Rapid microbial diversification in the oral and intestinal niches commenced shortly after hatching, with the relative abundances of dominant bacterial phyla, families, and genera increasing significantly in these two sites by day 10 post-hatching (Figure 1). Among the dominant bacterial phyla (>3% mean relative abundance in the oral cavity and >2% in the intestine) observed in 56-day-old hatchlings, four of five oral and five of six intestinal phyla were already detected in pre-feeding individuals (days 10 and 15) (Figure 1A). The bacteria colonizing the oral cavity and intestine after hatching but before first feeding were primarily derived from the external environment (water and/or soil) (Figure 1). Bayesian source tracking revealed that microbial community assembly prior to the onset of exogenous feeding was both dynamic and site-specific (Figure 3A). At hatching (day 1), the pioneer microbiota was primarily derived from egg-associated sources rather than the external environment, highlighting the critical role of the maternal-egg interface in establishing the founding community. Divergent assembly patterns emerged by day 15. Oral microbiota assembly was increasingly driven by colonization from environmental sources, whereas intestinal assembly was characterized by a pronounced increase in unknown sources. This divergence indicates that significant host-specific niche filtering and internal microbial restructuring occur within the intestinal tract, even prior to exogenous feeding. Consequently, the early-life microbiota was not merely a passive reflection of environmental exposure but was actively shaped by both external input and intrinsic host-mediated selection. Quantitative analysis of community assembly processes further elucidated the underlying mechanisms of this successional shift. In 1-day-old hatchlings, the dominance of homogenizing dispersal (100% in oral and 46.7% in intestinal microbiota) provided a mechanistic basis for the close taxonomic resemblance to egg-associated sources (Figures 3A and 3B). This suggests that during the initial stage, high rates of microbial dispersal from the maternal-egg interface predominated over other ecological processes, a pattern consistent with findings in other reptiles, in which the egg environment serves as the primary vehicle for vertical microbiota transmission.^2^^,^^11^ However, as hatchlings developed, microbiota assembly transitioned from dispersal-dominated to being primarily governed by undominated processes (ecological drift) and deterministic processes. The increasing contribution of homogeneous selection (reaching 30.6–36.1% by day 56) supports the role of host-specific niche filtering. This suggests that early assembly is highly stochastic, but the host’s internal environment gradually imposes deterministic pressures that select for colonizing taxa. This process shapes the microbiota toward a more specialized and stable state, both before and after the onset of feeding. The divergence of initially similar oral and intestinal microbial communities from a common inoculum is a hallmark of early crocodile development. At hatching, the high similarity between niches was primarily due to homogenizing dispersal from egg-associated sources. However, this spatial homogeneity was transient. βNTI analysis indicated that deterministic processes strengthened with hatchling age, consistent with intensifying niche-specific filtering.^30^ For example, the oral cavity, exposed to oxygen and environmental fluxes, selects for microbial communities rich in aerobic or facultative taxa. In contrast, the anaerobic conditions, low pH, and digestive enzymes of the intestinal tract impose strong deterministic selection, thereby shaping the pioneer pool into a specialized and functionally adapted community.^31^^,^^32^

Despite structural differences between the oral and intestinal microbiota, dominant taxa (>3% mean relative abundance) from the phylum to the genus level remained relatively stable between days 10 and 15 post-hatching, particularly in the oral cavity (Figures 1 and 4). The composition of core bacterial taxa in the oral and intestinal microbiota, particularly at the genus level, underwent substantial changes by day 56 compared to pre-feeding hatchlings (days 10 and 15) (Figures 4 and 5). Bacteria newly colonizing the oral cavity and intestine after the onset of feeding were primarily derived from ingested food, which explains the clear separation in the CCA between pre- and post-feeding microbiota based on the top 20 abundant ASVs (Figure 6B). Such diet-mediated microbial restructuring has been documented across all major vertebrate lineages, from fish^33^^,^^34^ and amphibians^35^^,^^36^^,^^37^ to reptiles,^38^^,^^39^ birds,^13^^,^^40^ and mammals.^41^^,^^42^ Importantly, accumulating evidence indicates that shifts in the gut microbiota are tightly coupled to distinct host developmental states, facilitating functional adaptations even in resource-limited environments. For instance, in holometabolous insects, cyclical expansion and contraction of gut microbial communities, linked to life stage transitions, orchestrate stage-specific ecological and metabolic adaptations during ontogeny.^43^ In cave-dwelling tadpoles, the gut microbiota adapts to limited resources by enhancing fibrolytic and fermentative capacity, a process that coincides with host developmental changes and a reduced growth rate.^37^ Similarly, in the common ostrich (*Struthio camelus*), taxonomic shifts in the gut bacterial community during early life coincide with yolk absorption and dietary transition.^13^

Third, environmental correlates of the microbiota are more pronounced in the oral cavity than in the intestine. Gram-negative bacteria of the genus *Achromobacter* that do not ferment lactose are widespread in both water and soil.^44^ In this study, *Achromobacter* was one of the most abundant genera not only in water and soil (Figure 1C), but also persisted in the oral cavity throughout the first 56 days of life (Figure 4). *Achromobacter* was one of the most abundant genera in the intestine of 1-, 10-, and 15-day-old hatchlings but decreased dramatically in 56-day-old hatchlings (Figure 4). We speculate that this temporal pattern aligns with two putative functions of *Achromobacter* bacteria: degrading yolk and other relatively simple polysaccharides to supply fermentable substrates for other gut microbes; degrading yolk anti-nutrients that bind minerals to release inorganic phosphorus and other essential minerals required by hatchlings.^45^ Notably, intestinal *Achromobacter* bacteria did not co-vary with their environmental (water and soil) congeners (Figure 5). Core nodes in the intestinal microbial co-occurrence networks of 10- and 15-day-old hatchlings were genera such as *Sphingobacterium*, *Stenotrophomonas*, *Staphylococcus*, and *Chloroplast* that were less abundant than *Achromobacter* in the environment (Figure 5). Furthermore, core intestinal microbial taxa showed limited overlap with co-occurrence network nodes (Figures 4 and 5). Collectively, these findings indicate that despite nonrandom post-hatching bacterial colonization at both sites, oral microbiota more closely reflect environmental input than intestinal microbial communities in *C. siamensis*. The genus-level divergence between intestinal and environmental microbiota in pre-feeding (10- and 15-day-old hatchlings) individuals suggests active selection for functionally critical bacteria, as observed in other animals.^8^^,^^18^^,^^29^^,^^46^ Of the six dominant (>3% mean relative abundance) oral bacterial genera (*Achromobacter*, *Comamonas*, *Deinococcus*, *Chryseobacterium*, *Alkanindiges*, and *Aeromonas*) in 56-day-old hatchlings, three (*Achromobacter*, *Alkanindiges*, and *Aeromonas*) were also dominant in the oral cavity of 10- and 15-day-old hatchlings.

Fourth, dietary correlates of the intestinal microbiota are more pronounced in the intestine than in the oral cavity. None of the seven dominant (>3% mean relative abundance) intestinal bacterial genera (*Deinococcus*, *Paracoccus*, *Nocardioides*, *Vagococcus*, *Luteimonas*, *Romboutsia*, and *Lactococcus*) in 56-day-old hatchlings was detected or dominant in pre-feeding hatchlings. Critically, all dominant intestinal genera detected at day 56 post-hatching emerged post-feeding, underscoring diet as a primary driver of intestinal microbiota assembly. These comparisons lead to the conclusion that dietary correlates of the microbiota are more pronounced in the intestine than in the oral cavity in *C. siamensis*.

Fifth, bacteria colonizing the oral cavity and/or intestine prior to the onset of exogenous feeding possess diverse functional potentials, including putative pathways associated with pesticide degradation, nitrogen fixation, cellulolysis, chitinolysis, amylolysis, and antioxidant activities, as well as bioremediation, which may be beneficial for *C. siamensis* hatchlings. The 12 most abundant genera in the oral cavity and/or intestine of 15-day-old hatchlings, listed in descending order by relative abundance, were: *Achromobacter*, *Staphylococcus*, *Stenotrophomonas*, *Elizabethkingia*, *Pseudomonas*, *Alkanindiges*, *Delftia*, *Sphingobacterium*, *Enterobacter*, *Corynebacterium*, *Flavobacterium*, and *Aeromonas* (Figure 4). Members of these dominant genera encompass lineages documented in the literature as pathogenic, nonpathogenic, or potentially probiotic. For example, members of five genera (*Staphylococcus*,^47^^,^^48^^,^^49^ *Elizabethkingia*,^50^^,^^51^ *Alkanindiges*,^52^ *Flavobacterium*,^53^ and *Aeromonas*^54^^,^^55^^,^^56^^,^^57^) include species or strains recognized as opportunistic pathogens, which may pose health risks under specific conditions. The genus *Flavobacterium* may confer beneficial effects through bioremediation, inferred from its documented involvement in organic matter cycling (e.g., uptake, degradation, and decomposition).^58^^,^^59^^,^^60^ The remaining seven dominant genera are frequently associated with putative beneficial roles. For example, *Achromobacter* spp. have been reported to confer host resistance to pesticides (e.g., in the red flour beetle *Tribolium castaneum*^61^) and to degrade plant cellulose (e.g., in the yak *Bos grunniens*^62^). Bacteria of the genus *Stenotrophomonas* may be considered potential symbionts due to cellulolytic and nitrogen-fixing capabilities^63^^,^^64^^,^^65^^,^^66^^,^^67^^,^^68^; however, their net impact on the host is highly context-dependent, varying with environmental and host physiological conditions. Importantly, functional roles can differ substantially among species within the same genus. Therefore, these taxonomic assignments primarily suggest the putative ecological potentials of early colonizers and do not denote fixed physiological attributes or guaranteed symbiotic outcomes. Bacteria of the genus *Pseudomonas* can be essential in protecting hosts from fungal and bacterial infections.^69^^,^^70^^,^^71^ Bacteria of the genus *Delftia* play key roles in organic matter degradation,^72^ while bacteria of the genus *Corynebacterium* can help combat oxidative stress and maintain intestinal homeostasis in hosts.^73^^,^^74^ Bacteria of the genus *Enterobacter* can provide hosts with benefits such as nutrient provisioning and degradation, as well as pathogen protection.^75^ Bacteria of the genus *Sphingobacterium* are noted for resistance to heavy metals and antibiotics,^76^^,^^77^ with some members exhibiting unique physiological traits such as biosurfactant production and ginsenoside-converting activity.^78^ Among these seven genera with putative beneficial roles, five (*Achromobacter*, *Stenotrophomonas*, *Delftia*, *Sphingobacterium*, and *Corynebacterium*) were also among the dominant taxa in the oral cavity and/or intestine of 10-d-old hatchlings (Figure 4). This finding indicates that key putatively functional microbial taxa are largely established by day 10 post-hatching, forming a foundational community structure prior to exogenous feeding. The ontogenetic shifts in predicted microbial functions over the first 56 post-hatching days further support that the establishment of functionally important oral and intestinal bacterial communities in hatchling *C. siamensis* prior to exogenous feeding (Figure 9).

In summary, this study yields the following key conclusions. First, egg-associated microbes serve as the primary inoculum for the early-life oral and intestinal microbiota of *C. siamensis*. This is supported by the close match in taxonomic composition and relative abundance of core bacterial taxa between the egg environment and the oral cavity/intestinal tract of 1-day-old hatchlings. Second, the oral and intestinal bacterial communities undergo rapid post-hatching diversification, as indicated by substantial increases in the relative abundances of dominant phyla, families, and genera exhibiting by day 10. Third, bacterial colonization in the oral cavity and intestine is non-random and niche-specific. This is evidenced by (1) the low prevalence of dominant oral and intestinal ASVs in environmental samples, and (2) the lack of significant correlations between the oral and intestinal bacterial communities throughout the 56-day post-hatching period. Fourth, the oral and intestinal bacterial communities established prior to exogenous feeding encode a broad spectrum of predicted functional potentials. Specifically, in 15-day-old hatchlings, the microbiota harbors taxa linked to diverse predicted functions, including metabolic activities, stress resistance, and environmental processes. Collectively, these findings support our hypothesis that the initial assembly of key microbial taxa in the oral cavity and intestinal tract occurs prior to residual yolk depletion.

### Limitations of the study

We identify three key limitations of this study. First, microbial functions were predicted from taxonomic profiles and published data, not experimentally verified. Second, dietary influences were deduced by comparing pre- and post-feeding groups, with no direct profiling of feed microbiota. Third, our samples comprised unhatched eggs and newly hatched hatchlings; non-invasive sex identification was unfeasible at this stage, so sex was excluded as a factor in community analyses. Follow-up studies across reptilian species with divergent residual yolk stores will test how widely this developmental microbial model applies.

## Resource availability

### Lead contact

Further information and requests for resources should be directed to and will be fulfilled by Dr. Xiang Ji (xji@wzu.edu.cn).

### Materials availability

This study did not generate new unique reagents.

### Data and code availability

• All data reported in this paper will be shared by the lead contact upon request.
• All 16S rRNA gene sequences obtained in this study have been deposited in the National Genomics Data Center (NGDC) GSA database (accession number CRA017394; BioProject PRJCA019895; Biosamples SAMC3881427-SAMC3881334).
• Any additional information required to reanalyze the data reported in this paper is available from the lead contact upon reasonable request.

## Acknowledgments

This study was supported by grants from the Financial Science and Technology Project of Hainan Province (grant no. ZDKJ2016009-1-2) and Hainan Key Laboratory of Herpetological Research (grant no. HKLHR202002). The authors would like to thank De-Fu Wang and Xin-Rong Zhang for help in sample collection during the research.

## Author contributions

X.J., Y.-F.Q., Y.D., and J.-Q.C.: conceived and designed the experiment; X.J., Y.-F.Q., and Y.D.: supervised the study; J.-Q.C., D.L., Q.-Y.M., Y.-T.Y., C.-X.L., Y.-H.S., and Y.D.: conducted the experiment and collected the data; J.-Q.C., D.L., and Y.-F.Q.: analyzed the data; X.J., Y.-F.Q., and J.-Q.C. wrote the paper. All authors contributed critically to the drafts and gave final approval for the manuscript.

## Declaration of interests

The authors declare no competing interests.

## STAR★Methods

### Key resources table

**Table:** 

REAGENT or RESOURCE	SOURCE	IDENTIFIER
**Biological samples**		

*Crocodylus siamensis* eggs	This paper	NA

**Critical commercial assays**		

Sterile cotton swabs	BKMANLAB	NA
Parafilm®M	Bemis	NA
EZNA® soil DNA kits	Omega Bio-tek	NA
AxyPrep DNA gel extraction kit	Axygen Biosciences	NA
NanoDrop2000	Thermo Fisher Scientific	NA
ABI GeneAmp®9700 thermocycler	ABI	NA
Illumina MiSeq PE300 platform	Illumina	NA

**Deposited data**		

DNA-seq data	This paper	GSA: CRA017394

**Software and algorithms**		

QIIME2	Bolyen et al.^79^	https://qiime2.org/
DADA2	Callahan et al.^80^	https://bioconductor.org/packages/release/bioc/html/dada2.html
SILVA 138 SSURef NR99 database	Bokulich et al.^81^	https://docs.qiime2.org/2023.9/data-resources/
LEfSe	Segata et al.^82^	http://galaxy.biobakery.org/
R version 4.4.1	R Software Foundation	https://www.r-project.org/
SourceTracker2 2.0.1	Knights et al.^83^	https://github.com/danknights/sourcetracker/releases
psych	Revelle^84^	https://cran.r-project.org/web/packages/psych/index.html
Gephi 0.10	Delgado-Baquerizo et al.^85^	https://gephi.org/
PICRUSt2	Douglas et al.^86^	https://picrust.github.io/picrust/

### Experimental model and study participant details

Our experimental models were Siamese crocodile (*Crocodylus siamensis*) eggs and hatchlings. All procedures involving live samples (eggs and hatchlings) complied with Chinese animal welfare regulations and were approved by the Animal Research Ethics Committee of Hainan Tropical Ocean University (IACUC 20220266).

We collected 10 clutches of eggs in mid-May 2022 from Longze Agricultural Science and Technology Development Co., Ltd., a commercial farm in Hainan, China, with official approval from the Hainan Provincial Department of Agriculture and Rural Affairs. Eggs were labelled individually and placed into foam boxes (520 × 370 × 160 mm) filled with sterilized vermiculite (autoclaved at 180 °C for 12 h before use) adjusted to a water potential of –50 kPa (1 g dried vermiculite: 1.7 g water)^87^ throughout incubation. Incubation proceeded at a constant laboratory temperature of 31 ± 0.3°C until hatching. Post-hatching individuals were housed in an outdoor aquaculture facility (3.5 × 3 × 0.7 m) lined with soil and fitted with a water channel (1.6 × 0.5 × 0.1 m). Facility ambient temperatures fluctuated 28–34°C (mean 31.5°C); evaporated water was replenished daily. First feeding occurred at 16 days post-hatching, timed to coincide with projected residual yolk exhaustion. Hatchlings were then offered framed tilapia (*Oreochromis mossambicus* and *O. niloticus*) every second day.

### Method details

#### Sample collection

Ten hatchlings (one per clutch) were randomly selected. On the day of hatching, microbial samples were collected from these 1-day-old individuals using sterile cotton swabs (BKMANLAB, China) from the oral cavity, albumen, inner eggshell, and intestinal tract (cloaca and posterior large intestine; hereafter ‘intestine’), with follow-up oral and intestinal sampling of the same individuals conducted on post-hatching days 10, 15, and 56.

Ten hatchlings were initially selected for microbial analysis. The final number of qualified samples was lower due to the exclusion of samples that failed to meet quality control criteria, such as insufficient biomass, low DNA yield, or poor sequencing quality. The final qualified sample set comprised: oral and intestinal samples from six 1-day-old, seven 10-day-old, ten 15-day-old, and nine 56-day-old hatchlings; albumen and eggshell samples from six hatched eggs. Environmental samples – three water (≈ 20 mm depth) and three soil (surface) samples – were collected on days 10, 15, and 56 post-hatching. This environmental sampling strategy was adopted to capture the microbial communities of both microhabitats (water and soil) that hatchlings frequently occupied. Prior to sampling each hatchling, the buccal cavity and cloaca were rinsed with sterile water to remove superficial contaminants. Oral (buccal cavity and gum lining) and intestinal (cloaca and posterior large intestine; 10–15 mm sampling depth) samples were collected using sterile swabs. This sampling depth was chosen to access the distal posterior large intestine (coprodeum), thereby minimizing contamination from the urogenital tract and external environment and ensuring that the microbial profile represented the core intestinal community. Each swab was placed in a 50 ml sterile conical tube containing 10–20 ml of sterile saline, pencil-labeled, and sealed with Parafilm®M (Bemis, USA). All samples were stored at −80 °C until further processing.

#### DNA extraction, PCR amplification, and sequencing

We used EZNA® soil DNA Kits (Omega Bio-tek, Norcross, GA, USA) to extract microbial genomic DNA from 94 samples according to the manufacturer’s instructions, verified DNA integrity by 1% agarose gel electrophoresis, and quantified DNA concentration via NanoDrop2000 (Thermo Fisher Scientific, Waltham, MA, USA). The V3-V4 hypervariable region of the 16S rRNA gene was amplified with barcoded primers 338F (5′-ACTCCTACGGGAGGCAGCAG -3′) and 806R (5′-GGACTACHVGGGTWTCTAAT-3′) on an ABI GeneAmp®9700 thermocycler (ABI, CA, USA). PCR reactions (20 μl) contained: 4 μl 5× TransStart FastPfu buffer (TransGen Biotech, Beijing, China), 2 μl 2.5 mM dNTP, 0.8 μl each of 5 μM primers, 0.4 μl TransStart FastPfu DNA polymerase (TransGen Biotech, Beijing, China), 10 ng template DNA, and ddH_2_O. PCR thermal cycling conditions were set as follows: initial denaturation at 95 °C for 3 min, followed by 27 cycles of denaturation at 95 °C for 30 s, annealing at 55 °C for 30 s, extension at 72 °C for 45 s, and final extension at 72 °C for 10 min. PCR products were purified with AxyPrep DNA Gel Extraction Kit (Axygen Biosciences, Union City, CA, USA), and equimolar amplicons were paired-end sequenced (2 × 300 bp) on an Illumina MiSeq PE300 platform (Illumina, San Diego, USA) according to the standard protocols of Majorbio Bio-Pharm Technology (Shanghai, China).

#### Microbial source tracking and community assembly

To quantify the relative contributions from putative microbial sources to the oral and intestinal microbiota, we conducted Bayesian source tracking using SourceTracker2 2.0.1.^83^ For this analysis, samples from the initial egg-associated environment (pooled albumen and inner eggshell, representing the environmental exposure of 1-day-old hatchlings) and subsequent environmental (water and soil) were designated as potential ‘source’ communities, whereas oral and intestinal samples were classified as ‘sink’ communities. Source communities were rarefied to the minimum sequence depth, and sink communities were rarefied to 1000 sequences per sample to maintain consistency across age groups. The Dirichlet prior parameters were assigned default values (*α*_1_ = 0.001, *α*_2_ = 0.1). To provide a phylogenetic basis for null model analysis, a rooted phylogenetic tree was constructed using the QIIME2 pipeline.^79^ Briefly, representative sequences were aligned with MAFFT, and ambiguous positions were filtered. A maximum-likelihood phylogenetic tree was then inferred using FastTree 2 under the GTR (+CAT) substitution model, followed by midpoint rooting. Community assembly mechanisms were categorized into deterministic and stochastic processes based on null model analysis.^32^^,^^88^ The beta-nearest taxon index (βNTI) was calculated to assess phylogenetic turnover, with ∣βNTI∣ > 2 indicating deterministic selection (either homogeneous or heterogeneous selection). Stochastic processes were identified when ∣βNTI∣ < 2 and were further categorized using the Raup-Crick metric based on the Bray-Curtis dissimilarity (RC_bray_) to distinguish among homogenizing dispersal (RC_bray_ < −0.95), dispersal limitation (RC_bray_ > 0.95), and undominated processes (∣RC_bray_∣ < 0.95).^32^^,^^88^

#### Temporal dynamics

We constructed AIC-optimized linear or non-linear regression models to describe phylum-level microbial (> 1% relative abundance) dynamics in the oral cavity and intestine over the first 56 post-hatching days, thereby ensuring the robustness of the fitted successional trends. We used the species-time relationship (STR) to construct a logarithmic linear model of bacterial richness over time.^89^ STR captures the dynamic changes in bacterial richness over time, where a greater exponent indicates a more rapid increase in species richness.^90^ We used the time-decay relationship (TDR) to evaluate the variation tendency of microbial similarity over time.^90^ The TDR equation is written as log_10_ (S) = constant − wlog_10_ (T), where S, T, and w represent the pairwise similarity in the microbial structure, the time interval and the change or turnover rate of the microbial community, respectively. We used Bray-Curtis distance to perform STR and TDR analyses because the distance effectively reflected the temporal changes in microbial richness and composition in the oral cavity and intestine in the first 56 post-hatching days. We used PICRUSt2 to predict the Kyoto Encyclopedia of Genes and Genomes (KEGG) functions from 16S data.^86^

### Quantification and statistical analysis

Raw sequences were processed in Quantitative Insights into Microbial Ecology 2 (QIIME2)^79^ for demultiplexing, quality filtering, and primer removal. DADA2 denoised, merged, and generated ASVs.^80^ Taxonomy was assigned against SILVA 138 SSURef NR99 via q2-fragment classifier.^81^ ASVs < 0.005% relative abundance or present in only one sample were excluded. Abundances were rarefied to the minimum sample depth. Core ASVs (> 2% mean relative abundance in ≥ 90% of all oral or intestinal samples per age group^91^) were analyzed for the site (oral cavity versus intestine) and age (days 1, 10, 15, 56 post-hatching) effects.

ASVs > 0.1% relative abundance were retained for network analysis to reduce spurious correlations and focus on stable community interactions.^92^ We used the R ‘psych’ package^84^ to calculate Spearman correlations between the environmental microbiota and the oral or intestinal microbiota and identified co-occurring ASVs (|*r*| > 0.6, *p* < 0.001).^85^ Networks were visualized in Gephi 0.10,^93^ with six topology parameters (average degree, average path length, modularity, diameter, density, and average clustering coefficient) quantifying environmental correlates of the oral and intestinal microbiota in early post-hatching days. We used canonical correspondence analysis (CCA) to evaluate contributions of the top 20 ASVs to microbial community variation in the oral cavity and intestine.

For β-diversity, we calculated both Bray-Curtis (compositional) and weighted UniFrac (phylogenetic) distances. We then performed PCoA, ANOSIM, and PERMANOVA (999 permutations) to test community structure differences between samples from hatchlings at different days of age and between oral and intestinal samples from hatchlings at the same age. We performed linear discriminant analysis effect size (LEfSe) and linear discriminant analysis (LDA)^82^ to identify differentially abundant taxa (from phylum to genus; log LDA scores > 4) in the oral cavity and intestine in the Galaxy Biobakery platform, using Mann-Whitney test to confirm significance.

All descriptive statistics were expressed as mean ± standard error (SE), and the significance level was set at *p* = 0.05. We used Mantel test to assess correlations between the environmental microbiota and oral or intestinal microbiota across age groups. We used QIIME2 to calculate three α-diversity indices, community richness (ACE index), community diversity (Shannon entropy index) and community evenness (Pielou evenness index). We used Kruskal-Wallis test, dependent (paired) sample *t* test, and independent sample *t* test to examine whether microbial α-diversity differed among samples collected from hatchlings at different days of age, between oral and intestinal samples collected from hatchlings at the same sampling time point, and between water and soil samples, respectively.
