# Supplementary material for: Residual yolk depletion matches the establishment of functional oral-intestinal microbiota in hatchling Siamese crocodiles
Source: iScience. 2026 Aug 4;29(8):117047. doi: 10.1016/j.isci.2026.117047 (PMC13463808; doi:10.1016/j.isci.2026.117047)
Supplement: Supplementary file 1 [file mmc1.pdf]

iScience, Volume *29*

Supplemental information

Residual yolk depletion matches the establishment of functional oral-intestinal microbiota in hatchling Siamese crocodiles

Jun-Qiong Chen, Dan Liu, Qing-Yan Meng, Yun-Tao Yao, Chi-Xian Lin, Ying-Hao Shi, Yu Du, Yan-Fu Qu, and Xiang Ji

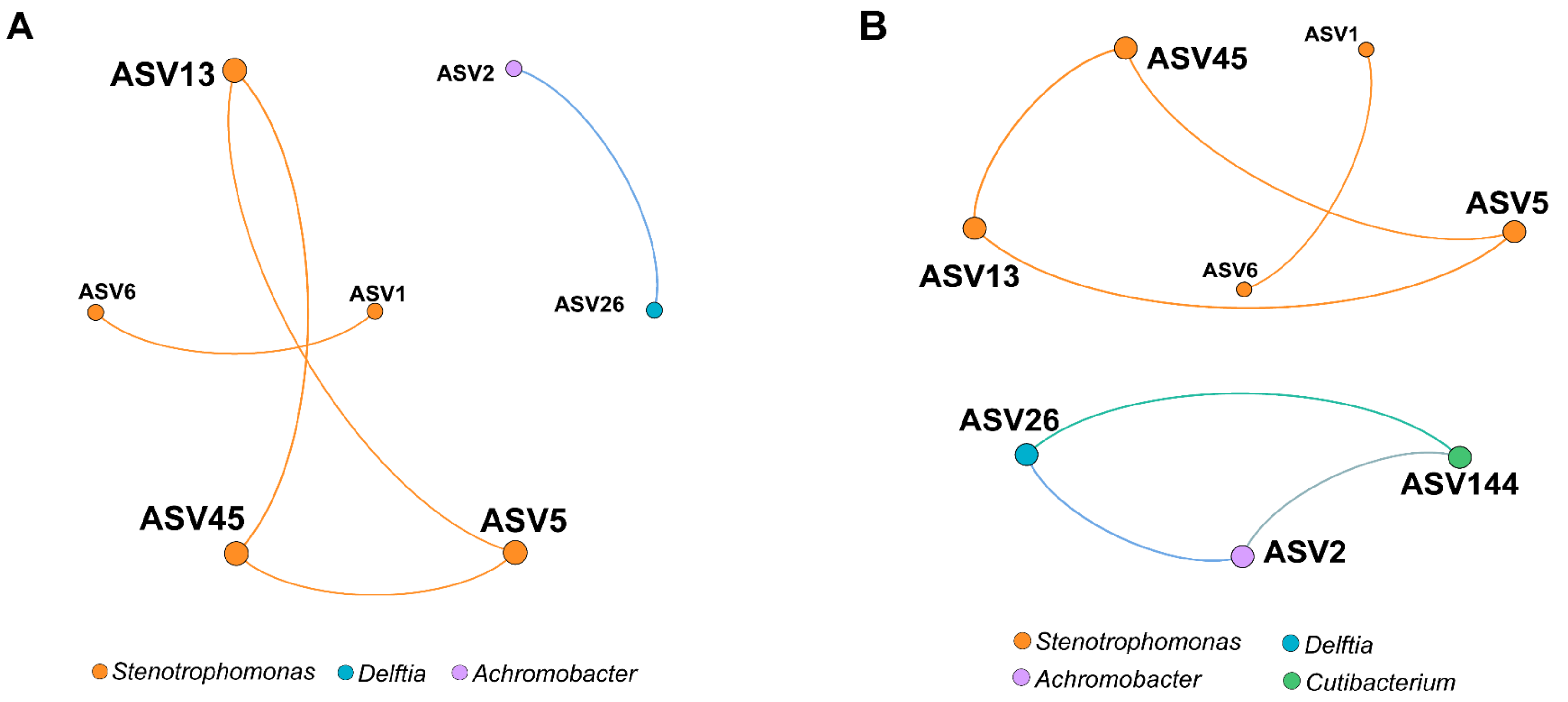

Figure S1. Co-occurrence networks of oral and intestinal microbiota and microbiota in eggs in newborn hatchlings
(A) Oral microbiota. (B) Intestinal microbiota. ASVs with a mean relative abundance > 1% were included. Nodes were colored according to microbial genera, and node size represented the correlation between ASVs. Information on key genera was below the network figures

Table S1. Statistical analysis results of microbial α-diversity

Samples compared	ACE	Pielou Evenness	Shannon-Wiener
Eggshell_Albumen*	$t = 0.58$, $df = 5$, $P = 0.58$	$t = 0.01$, $df = 5$, $P = 0.99$	$t = 0.77$, $df = 5$, $P = 0.47$
Soil: 10_15_56	$H_{2,9} = 1.16$, $P = 0.56$	$H_{2,9} = 3.82$, $df = 2$, $P = 0.15$	$H_{2,9} = 5.96$, $df = 2$, $P = 0.05$
Water: 10_15_56	$H_{2,9} = 5.60$, $P = 0.06$	$H_{2,9} = 1.42$, $df = 2$, $P = 0.49$	$H_{2,9} = 5.60$, $df = 2$, $P = 0.06$
1O_1I*	$t = 3.62$, $df = 5$, $P < 0.02$; I > O	$t = 1.142$, $df = 5$, $P = 0.31$	$t = 2.79$, df = 5, $P = 0.04$; I > O
10O_10I*	$t = 0.71$, $df = 6$, $P = 0.50$	$t = 2.75$, $df = 6$, $P = 0.03$; I < O	$t = 2.04$, $df = 6$, $P = 0.09$
15O_15I*	$t = 2.33$, $df = 9$, $P < 0.05$; I > O	$t = 0.06$, $df = 9$, $P = 0.95$	$t = 0.57$, $df = 9$, $P = 0.58$
56O_56I*	$t = 1.71$, $df = 8$, $P = 0.13$	$t = 1.79$, $df = 8$, $P = 0.11$	$t = 0.25$, $df = 8$, $P = 0.81$
1O_10O_15O_56O	$H_{3,32} = 23.12$, $P < 0.01$	$H_{3,32} = 20.118$, $P < 0.01$	$H_{3,32} = 22.52$, $df = 3$, $P < 0.01$
1I _10I _15I _56I	$H_{3,32} = 14.01$, $P < 0.01$	$H_{3,32} = 20.155$, $P < 0.01$	$H_{3,32} = 20.03$, $df = 3$, $P < 0.01$
1O_Egg	$t = 2.02$, $df = 16$, $P = 0.06$	$t = 1.3$, $df = 16$, $P = 0.90$	$t = 0.53$, $df = 16$, $P = 0.61$
1I_Egg	$t = 5.00$, $df = 16$, $P < 0.01$; I > Egg	$t = 0.16$, $df = 16$, $P = 0.88$	$t = 1.39$, $df = 16$, $P = 0.18$
10O_Water	$t = 2.69$, $df = 14$, $P = 0.02$; O < Water	$t = 4.19$, $df = 14$, $P < 0.01$; O < Water	$t = 3.83$, $df = 14$, $P = 0.02$; O < Water
10O_Soil	$t = 6.02$, $df = 14$, $P < 0.01$; O < Soil	$t = 3.68$, $df = 14$, $P = 0.02$; O < Soil	$t = 5.13$, $df = 14$, $P < 0.01$; O < Soil
10I_Water	$t = 1.74$, $df = 14$, $P = 0.10$	$t = 4.08$, $df = 14$, $P < 0.01$; I < Water	$t = 3.78$, $df = 14$, $P < 0.01$; I < Water
10I_Soil	$t = 5.55$, $df = 14$, $P < 0.01$; I < Soil	$t = 4.33$ $df = 14$, $P = 0.02$; I < Soil	$t = 5.22$, $df = 14$, $P < 0.01$; I < Soil
15O_Water	$t = 5.13$, $df = 17$, $P < 0.01$; O < Water	$t = 4.12$, $df = 17$, $P < 0.01$; O < Water	$t = 5.12$, $df = 17$, $P < 0.01$; O < Water
15O_Soil	$t = 8.10$, $df = 17$, $P < 0.01$; O < Soil	$t = 4.58$, $df = 17$, $P < 0.01$; O < Soil	$t = 6.71$ $df = 17$, $P < 0.01$; O < Soil
15I_Water	$t = 2.31$, $df = 17$, $P = 0.03$; I < Water	$t = 3.41$, $df = 17$, $P < 0.01$; I < Water	$t = 3.23$, $df = 17$, $P < 0.01$; I < Water
15I_Soil	$t = 6.73$, $df = 17$, $P < 0.01$; I < Soil	$t = 4.03$, $df = 17$, $P < 0.01$; I < Soil	$t = 5.15$, $df = 17$, $P < 0.01$; I < Soil
56O_Water	$t = 0.44$, $df = 16$, $P = 0.67$	$t = 0.80$, $df = 16$, $P = 0.43$	$t = 0.33$, $df = 16$, $P = 0.74$
56O_Soil	$t = 5.07$, $df = 16$, $P < 0.01$; O < Soil	$t = 2.11$, $df = 16$, $P = 0.05$; O < Soil	$t = 3.43$, $df = 16$, $P < 0.01$; O < Soil
56I_Water	$t = 1.88$, $df = 16$, $P = 0.08$	$t = 1.26$, $df = 16$, $P = 0.28$	$t = 0.12$, $df = 16$, $P = 0.91$
56I_Soil	$t = 6.39$, $df = 16$, $P < 0.01$; O < Soil	$t = 1.23$, $df = 16$, $P = 0.26$	$t = 3.69$, $df = 16$, $P < 0.01$; O < Soil

Results of the Kruskal-Wallis test, dependent (paired) sample t-test (with *), and independent sample t-test (without *) for microbial α-diversity. O and I indicate oral and intestinal microbial samples, respectively. 1O, 10O, 15O, and 56O represent oral samples respectively collected from 1-d-old, 10-d-old, 15-d-old, and 56-d-old hatchlings; 1I, 10I, 15I, and 56I represent intestinal samples respectively collected from 1-d-old, 10-d-old, 15-d-old, and 56-d-old hatchlings

Table S2. Results of the Mann-Whitney test for 14 unique bacterial markers

Microbial sample	Bacterial taxon	$U_{n1,n2}$ values and significance levels
10I	g_*Brevundimonas*	$U_{7,57} = 368$, $P < 0.0001$
15I	c_Bacilli	$U_{10,54} = 431$, $P < 0.01$
15I	o_Enterobacterales	$U_{10,54} = 479$, $P < 0.0001$
15I	g_*Staphylococcus*	$U_{10,54} = 506$, $P < 0.0001$
15I	g_*Rhodococcus*	$U_{10,54} = 476.5$, $P < 0.0001$
15I	g_*Microbacterium*	$U_{10,54} = 401$, $P < 0.01$
15I	f_Enterobacteriaceae	$U_{10,54} = 467$, $P < 0.0001$
15I	f_Nocardiaceae	$U_{10,54} = 467.5$, $P < 0.0001$
56I	o_Rhodobacterales	$U_{9,55} = 413$, $P < 0.001$
56I	f_Rhodobacteraceae	$U_{9,55} = 413$, $P < 0.001$
56I	o_Lactobacillales	$U_{9,55} = 387$, $P < 0.01$
56I	g_*Paracoccus*	$U_{9,55} = 413$, $P < 0.001$
56I	o_Peptostreptococcales_Tissierellales	$U_{9,55} = 355.5$, $P < 0.05$
56O	f_Propionibacteriaceae	$U_{9,55} = 436$, $P < 0.0001$

56O represents oral samples collected from 56-d-old hatchlings; 10I, 15I, and 56I represent intestinal samples respectively collected from 10-d-old, 15-d-old, and 56-d-old hatchlings. Letters c, o, f, and g indicate class, order, family, and genus, respectively

Table S3. Network topological characteristics of oral/intestinal and environmental microbial communities

Group	Node counts	Edge counts	Average degree	Average weighted degree	Average path length	Average clustering coefficient	Modularity	Diameter	Density
1O_E	7	5	1.429	1.339	1	1	0.565	1	0.238
1I_E	8	7	1.750	1.624	1	1	0.604	1	0.250
10O_S	63	200	6.349	6.217	2.365	0.742	0.563	6	0.102
10O_W	60	152	5.067	4.882	2.209	0.760	0.721	6	0.086
10I_S	67	230	6.866	6.710	2.200	0.816	0.572	6	0.104
10I_W	55	116	4.218	4.114	2.282	0.832	0.640	6	0.078
15O_S	59	166	5.627	5.384	2.212	0.824	0.666	6	0.097
15O_W	74	221	5.973	5.622	2.420	0.749	0.640	6	0.082
15I_S	82	241	5.878	5.592	2.791	0.825	0.760	8	0.073
15I_W	89	243	5.461	5.167	2.549	0.791	0.713	7	0.062
56O_S	75	128	3.413	3.261	1.610	0.790	0.836	4	0.046
56O_W	67	144	4.299	4.110	2.759	0.744	0.755	7	0.065
56I_S	67	94	2.806	2.694	2.056	0.698	0.826	6	0.043
56I_W	84	216	5.143	4.932	2.643	0.721	0.675	8	0.062

1O, 10O, 15O, and 56O indicate oral samples respectively collected from 1-d-old, 10-d-old, 15-d-old, and 56-d-old hatchlings. 1I, 10I, 15I, and 56I indicate intestinal samples respectively collected from 1-d-old, 10-d-old, 15-d-old, and 56-d-old hatchlings. E, W, and S indicate environmental samples respectively collected from hatched eggs (albumen and inner eggshell), water, and soil
